# Multiview Locally Linear Embedding for Effective Medical Image Retrieval

**DOI:** 10.1371/journal.pone.0082409

**Published:** 2013-12-13

**Authors:** Hualei Shen, Dacheng Tao, Dianfu Ma

**Affiliations:** 1 State Key Laboratory of Software Development Environment, School of Computer Science and Engineering, Beihang University, Beijing, China; 2 Center for Quantum Computation and Intelligent Systems, Faculty of Engineering and Information Technology, University of Technology, Sydney, New South Wales, Australia; Institution of Automation, CAS, China

## Abstract

Content-based medical image retrieval continues to gain attention for its potential to assist radiological image interpretation and decision making. Many approaches have been proposed to improve the performance of medical image retrieval system, among which visual features such as SIFT, LBP, and intensity histogram play a critical role. Typically, these features are concatenated into a long vector to represent medical images, and thus traditional dimension reduction techniques such as locally linear embedding (LLE), principal component analysis (PCA), or laplacian eigenmaps (LE) can be employed to reduce the “curse of dimensionality”. Though these approaches show promising performance for medical image retrieval, the feature-concatenating method ignores the fact that different features have distinct physical meanings. In this paper, we propose a new method called multiview locally linear embedding (MLLE) for medical image retrieval. Following the patch alignment framework, MLLE preserves the geometric structure of the local patch in each feature space according to the LLE criterion. To explore complementary properties among a range of features, MLLE assigns different weights to local patches from different feature spaces. Finally, MLLE employs global coordinate alignment and alternating optimization techniques to learn a smooth low-dimensional embedding from different features. To justify the effectiveness of MLLE for medical image retrieval, we compare it with conventional spectral embedding methods. We conduct experiments on a subset of the IRMA medical image data set. Evaluation results show that MLLE outperforms state-of-the-art dimension reduction methods.

## Introduction

Medical image interpretation is a procedure which requires high accuracy. Currently, radiologists rely on both knowledge and heuristics to accomplish this procedure [1]. As a result of perceptual, training and fatigue differences among radiologists, there are variations in the interpretations made by different personnel to the same image [2]–[4]. Moreover, with the wide deployment of modern medical imaging devices in hospitals, large numbers of medical images are produced every day, placing an additional burden on radiologists. On one hand, they have to render accurate diagnoses for each image; on the other, they have to interpret large amounts of medical images within a limited time frame [4].

To tackle these challenges, content-based image retrieval (CBIR) has been introduced into the radiology interpretation routine in recent years [4]–[11]. CBIR employs visual descriptors to represent medical images, and machine learning techniques to retrieve and compare those images. For a given query image, the technique of contend based medical image retrieval (CBMIR) aims to find its visually similar and semantically relevant counterparts by retrieving samples from a given medical image archive. In the context of CBMIR, medical image is usually represented as vector with attributed features. Then similarity between two medical images is measured by distance between the corresponding feature vectors. This helps radiologists to efficiently extract similar cases from a variety of archives, thus providing assistance with medical image interpretation and decision making.

Similar to CBIR, CBMIR faces two basic issues: using discriminative visual features to represent medical images and assessing similarity among images represented in the feature space. This paper focuses on the former issue.

By contrast with images in other domains, most medical images have gray values, and fine details are emphasized in the image content [4]. A single feature therefore cannot cover all the details of a medical image. Following this observation, many visual features have been simultaneously employed to reveal different aspects of medical images. Dimitrovski et al. [12] extracted pixel value, local binary pattern (LBP) [13], edge histogram descriptor [14] and SIFT features [15] to represent medical images. Lehman et al. [16] proposed an automatic medical image categorization framework that combines four types of texture feature and one intensity feature to represent medical images. Chen et al. [17] extracted six textual features to represent ultrasound images. In [18], Wu et al. recently extracted texture features and morphological features to classify ultrasound breast tumor images. Moreover, Dy et al. [19] proposed a lung image retrieval method based on 110 features. For a detailed review of features used in the medical domain, please refer to [4]. In this paper, we call these visual features “multiview features”.

With the increasing use of multiview features, medical CBIR also suffers from the “curse of dimensionality”. To reduce the dimension of feature vectors, one conventional solution is to concatenate these feature vectors into a long vector, and then use traditional dimension reduction techniques, e.g., locally linear embedding (LLE) [20], principal component analysis (PCA) [21] or laplacian eigenmaps (LE) [22], to project the concatenated vector to a low-dimensional subspace. Huang et al. [23] built a computer-aided breast cancer diagnosis system using PCA to project original high-dimensional textual features into a low-dimensional feature space. Zhang et al. [24] proposed a brain midsagittal plane image recognition system that employed PCA to perform dimensionality reduction. Chen et al. [17] used PCA to reduce the dimension of textural feature vectors extracted from breast ultrasound images. In [25], Cho et al. employed linear discriminant analysis (LDA) to perform feature selection. Although these solutions have achieved promising results, there is room for performance enhancement, because these methods coarsely perform dimension reduction on all features and ignore the fact that different features have wide-ranging physical meanings. Recently, Bagci et al. [26] proposed a hybrid scheme for chest radiological image feature selection. They first selected features which could coarsely identify abnormal imaging patterns. Then they refined the selected features to enhance prediction accuracy.

To solve these problems, and considering the complementary properties of various features, we formulate a new method called multiview locally linear embedding (MLLE) to represent medical images in a low-dimensional feature space that is simultaneously learned from multiview features. MLLE is proposed in the context that multiview learning has received intensive attentions in the machine learning community [27]–[35]. The key idea of MLLE comes from patch alignment framework [36] and LLE. The patch alignment framework unifies discrete spectral analysis-based dimensionality algorithms in two stages: local patch construction and whole alignment [36]. LLE constructs a local patch in the low-dimensional space by preserving the patch’s linear reconstruction relation in original space, whereas MLLE constructs local patches from each feature space by preserving the geometric structure of patches according to the LLE criterion. To explore the complementary properties among multiview features, MLLE assigns various weights to patches from different feature spaces. Finally, MLLE uses global coordinate alignment [36], [37] and alternating optimization [38] techniques to learn a smooth low-dimensional embedding from the multiview features. We present a detailed evaluation of MLLE for CBMIR to demonstrate its effectiveness. Compared to conventional dimension reduction methods, e.g., PCA, LLE, LE, MLLE differs in the following ways: 1) MLLE uses LLE to obtain the optimal low-dimensional subspace on each view, and 2) MLLE learns a smooth low-dimensional global subspace by exploring complementary properties of each view.

To evaluate performance of the proposed MLLE, we conduct experiments on an IRMA [39] coded medical image data set [40]. IRMA medical image coding system [39] is a mono-hierarchical multi-axial classification standard for medical images. The system classifies medical images from four orthogonal axes: imaging modality, body orientation, examined body region and examined biological system. IRMA coding system is applicable to medical images obtained by different medical imaging techniques, which include computed tomography (CT), digital radiography (DR), magnetic resonance imaging (MRI), and positron emission tomography (PET), etc.

## Multiview Locally Linear Embedding

In this section, we detail the presented dimension reduction algorithm, i.e., MLLE. To better present MLLE, we first explain meanings of math notations used in this paper.

In the rest of this paper, 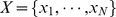 denotes medical image data set, which contains 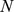 medical images. 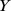 denotes the corresponding low-dimensional embedding of 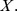 For each medical image 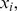 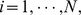 we extract 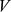 different low level features to represent its visual content. Then we say that 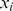 has 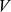 different views: 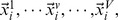 where 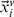 is the feature vector of 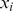 on the 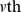 view. Accordingly, 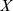 has 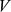 different views: 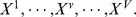 Where 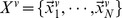 is the feature matrix of 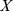 on the 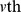 view. 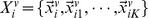 represents the local patch of 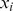 built on the 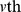 view, which contains 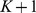 images. Where 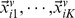 are 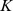 nearest neighbors of 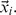 Detailed description of these math notations is listed in **Table 1**.

**Table 1:** Important notations used in this paper.

Notation	Description	Notation	Description
*X*	medical image dataset	*M*	local Gram matrix
*Y*	dimension-reduced medical image dataset	*M_kt_*	(*k*, *t*)th entry of matrix *M*
*N*	size of medical image dataset *X*	*M* ^−1^	inverse of matrix *M*
*X^v^*	feature matrix of *X* on *v*th view	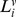	local patch optimization of 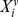
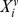	*i*th patch on *v*th view	*L^v^*	whole patch optimization of *X^v^*
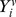	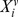 low-dimensional embedding of	*V*	number of multiview features
*x*	medical image contained in *X*	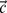	contribution vector
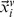	feature vector of *i*th image on *v*th view	*S_i_*	selection matrix
*m_v_*	dimension of *v*th feature space	*I*	identity matrix
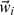	reconstruction coefficient vector in LLE	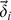	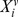 LLE reconstruction error in
*K*	number of nearest neighbors	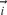	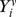 LLE reconstruction error in
*d*	dimension of *Y*	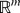	*m*-dimensional Euclidean space
*r*	scaling factor	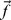	vector in Euclidean space

### Local Patch Construction

#### Local patch construction on single view

Given a point 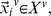 its local patch is defined as 
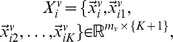
 where 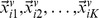 are 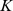 nearest neighbors of 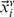 in 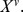 LLE preserves the local geometry of 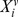 by assuming that 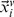 is reconstructed from 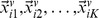 by linear coefficients [20]

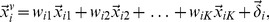
(1)where 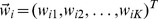 is determined by minimizing reconstruction error 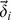




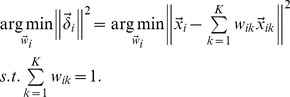
(2)By solving (2), we get
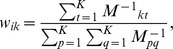
where 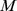 is a local Gram matrix, 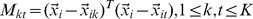 [20].

When 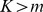 or when data points 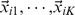 are not in general position, matrix 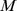 is singular or near singular [41]. To avoid this, a regularization term is added to each entry of 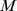 according to the following criterion [41]:
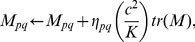
(3)where constant 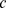 satisfies 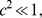 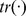 is the trace operator. And 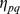 is defined as



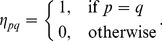
(4)LLE assumes 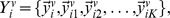 the corresponding local patch of 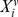 in the learned low-dimensional embedding, is also reconstructed by 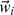


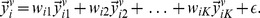
(5)


Similar to equation (2), 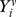 is determined by minimizing the reconstruction error 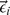


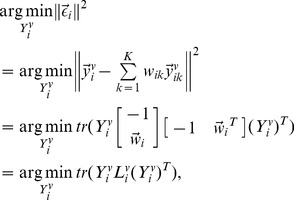
(6)where 
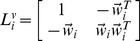
 encodes the local geometric information of 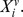


#### Local patch construction on multiple views

Each sample 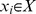 has different local patches on different views, i.e., 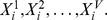 These multiview local patches correspond to various low-dimensional local patches. We denote these low dimensional local patches as 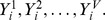 The differing features make different contributions to the representation of the medical image in the final low-dimensional embedding 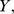 so these low-dimensional local patches have different degrees of importance in determining 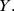 Considering this, we have the following objective function of multiview local patch optimization for the 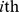 patch
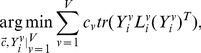
(7)where 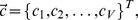 the 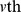 entry 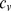 implies the contribution of 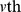 view to learn the final embedding 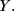


### Global Coordinate Alignment

For each local patch 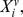 there is a low-dimensional embedding 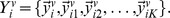 By assuming that all 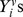 are chosen from the final embedding 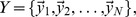 i.e., 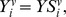 we can obtain the final low-dimensional embedding 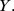 Selection matrix 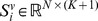 is defined as
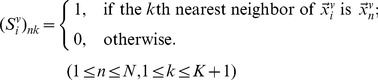
(8)


Considering the whole medical image data set 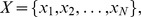 we can unify all local patches into the final embedding 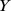 to obtain the global coordinate alignment (detailed derivation is given in Appendix S1)
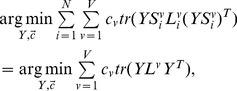
(9)where 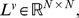, 
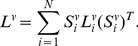
(10)


### Objective Function

To uniquely determine the low-dimensional embedding 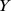 from (9), we add the constraint 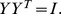 Thus 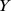 is obtained by solving the optimization problem
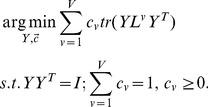
(11)


The solution to 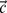 is 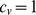 corresponding to the 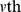 view which minimizes 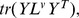 and 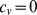 otherwise. This means that only one view is selected to learn the low-dimensional embedding 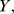 while other views are discarded. To avoid this, we set 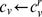 with 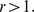 Then the optimization problem in (11) reduces to
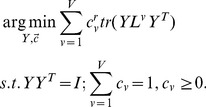
(12)


### Alternating Optimization

There are two unknown parameters, i.e., 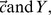 in (12). Here we employ the alternating optimization technique [38] to solve the optimization problem. The alternating optimization procedure includes the following two steps.

*Step 1: Fix* 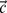 *to update* 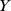


When 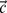 is fixed, the optimization problem in (12) equals
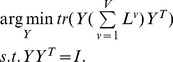
(13)


Because 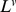 is symmetric and positive semidefinite (the proof is given in Appendix S2), then, 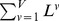 is symmetric and positive semi-definite. Hence, the optimization problem in (13) can be solved by using eigenvalue decomposition on 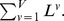 The globally optimal solution is the eigenvectors having the smallest 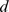 eigenvalues of 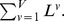


*Step 2: Fix* 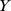 *to update* 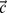


When 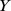 is fixed, the optimization problem in (12) can be solved by using Lagrange optimization. The Lagrange function is
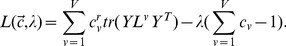
(14)


By taking the derivate of 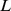 with respect to unknown parameter 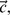 and given that 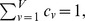


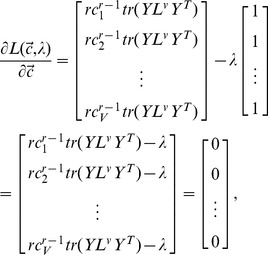
(15)we get
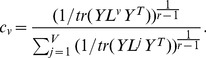
(16)


## Experiment Setup

In this section, we describe experiment setup for performance evaluation of MLLE for CBMIR. We organize this section as follows. In Section 3.1, we introduce our test bed, i.e., IRMA medical image data set. In Section 3.2, medical image feature extraction is detailed.

### IRMA Medical Image Data Set

**Figure 1:**
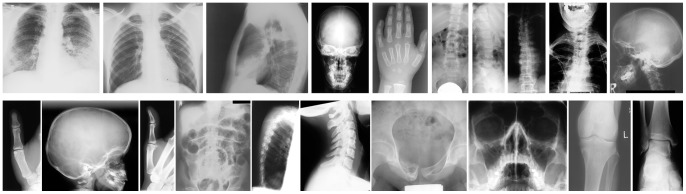
Example images from IRMA medical image data set. Each image belongs to a different category.

The IRMA medical image data set is a popular benchmark database used to evaluate CBMIR [6], [12], [42], [43]. The new version of the IRMA medical image data set [40] contains 193 categories with a total of 12,677 fully annotated gray value radiographs in a training set. These images are 8 bits per pixel. The images are categorized according to a mono-hierarchical multi-axial classification standard called IRMA coding system [39]. The coding system classifies a medical image from four orthogonal axes: imaging modality, body orientation, body region examined and biological system examined. We select the first 57 categories containing a total of 10,902 images from the training set for our experiment. **Figure 1** shows examples of the images used in our evaluation.

### Feature Extraction

All images in the IRMA dataset are gray value images, which encode ample texture information. We use three image descriptors, i.e., local binary patterns (LBP) [13], SIFT [15], and pixel intensity, to extract the visual features from each medical image.

To enhance the discriminability of the image descriptors, we divide the medical image into equal regions for each descriptor. In each region, an image descriptor is employed to extract the visual features. Finally, we concatenate all the feature vectors obtained from the regions in a single long vector to represent the image. For each image descriptor, we employ four different image division schemes. There are three image descriptors, and each image descriptor generates four different features. Thus, we obtain twelve different features from each image. The feature extraction procedures of each image descriptor are detailed below.

#### LBP

LBP is a powerful descriptor for analyzing two-dimensional textures. LBP has the advantages that it is robust to gray-scale variations and low computational complexity. This makes LBP appropriate for gray-scale medical image analysis.

Formally, for center pixel 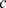 at 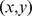 with gray value 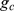, there are *P* equally spaced pixels contained in the circularly symmetric neighbor set of 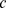 with radius 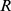. LBP assigns a unique value to the center pixel 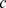 [13]:
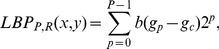
(17)where 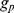 is the gray value of the 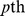 neighbor of center pixel 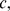




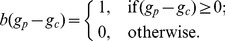
(18)Observing LBP value in binary circular representation, we find that a vast majority of LBP binary codes, sometimes more than 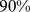, have “uniform” appearance [13]. Here, uniform appearance indicates that there are limited numbers of 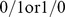 transitions in LBP code. These uniform binary patterns capture discriminant local features, e.g., edges, corners, and spots, of the image content. After computing LBP values over an examined image or image region pixel by pixel, these LBP values are accumulated into a discrete occurrence histogram. Uniform patterns in the histogram with different LBP values are accumulated to various bins, while the remaining “non-uniform” patterns are accumulated in another bin.

In our implementation, we use the 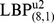 operator to compute the LBP values over a medical image, pixel by pixel. The subscript (8, 1) means that eight neighbors, equally contained in the circle with radius one, are utilized to determine the LBP value of the center pixel. Clearly, the resulting LBP value can be encoded into an eight bits binary string. The superscript u2 represents a uniform pattern which has at most two 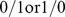 transitions. For an eight bits LBP binary string, there are 58 u2 patterns. Hence the resulting discrete occurrence histogram has 59 bins.

**Figure 2:**
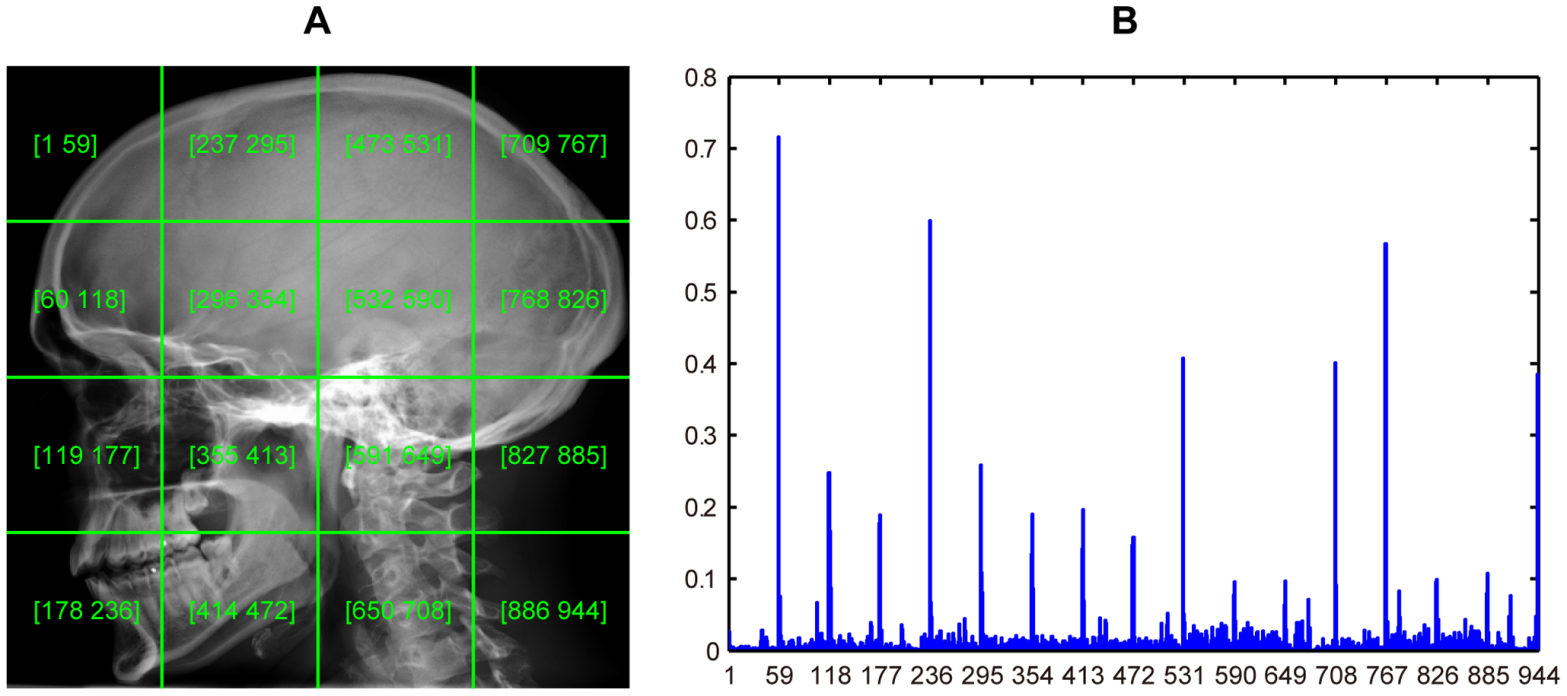
A medical image and its LBP histogram. (A) Image is equally divided into 4×4 regions. Text presented on each region is the coordinate interval of region LBP histogram in the concatenated histogram shown in (B). (B) Concatenated LBP histogram.

To enhance the discriminability of the LBP descriptor, we divide the medical image into equal regions. A normalized 59-bin histogram is built for each region. Finally, these normalized histograms are concatenated into a single histogram as a feature vector of the image. We employ four image division schemes: 3×3, 4×4, 5×5 and 6×6, giving us four different LBP feature vectors for each feature: 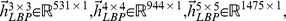 and 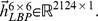 **Figure 2** demonstrates a 4×4 image division scenario and the concatenated LBP histogram extracted from the image.

#### SIFT

Following the bag of features paradigm [44] and dense sampling strategy, we build SIFT histograms to present medical images. We begin by extracting 128-D SIFT vectors [15] from patches densely sampled from the image. The sampling space and patch size are set as 8 and 16×16, respectively.

The next step is to build a visual word dictionary over all the SIFT vectors extracted from the entire data set. Following the settings in [12], we employ K-means clustering to learn the dictionary. Euclidean distance is used as the measurement of the distance between two SIFT vectors. To reduce computing time, we set the number of iterations as 100. The visual word dictionary size is set as 500. We finally acquire a SIFT visual word dictionary 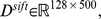 where each column vector 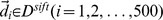 is the centroid SIFT vector generated by K-means clustering. We call column vector 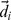 a “visual word”.

Via dense sampling, each sampled image region 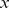 is represented as a collection of SIFT vectors 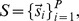 where P is the total number of patches sampled from 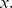 For each SIFT vector 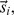 there exists a unique visual word 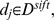 which is nearest to 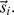 We assign the visual word index, i.e., 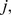 to 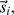 so that each patch sampled from 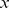 has a unique index in the visual word dictionary 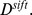 Consequently, 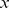 can be denoted as a collection of visual word indexes. Accumulating these indexes into a 500-bin histogram, we obtain a SIFT histogram 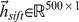 to present 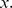


**Figure 3:**
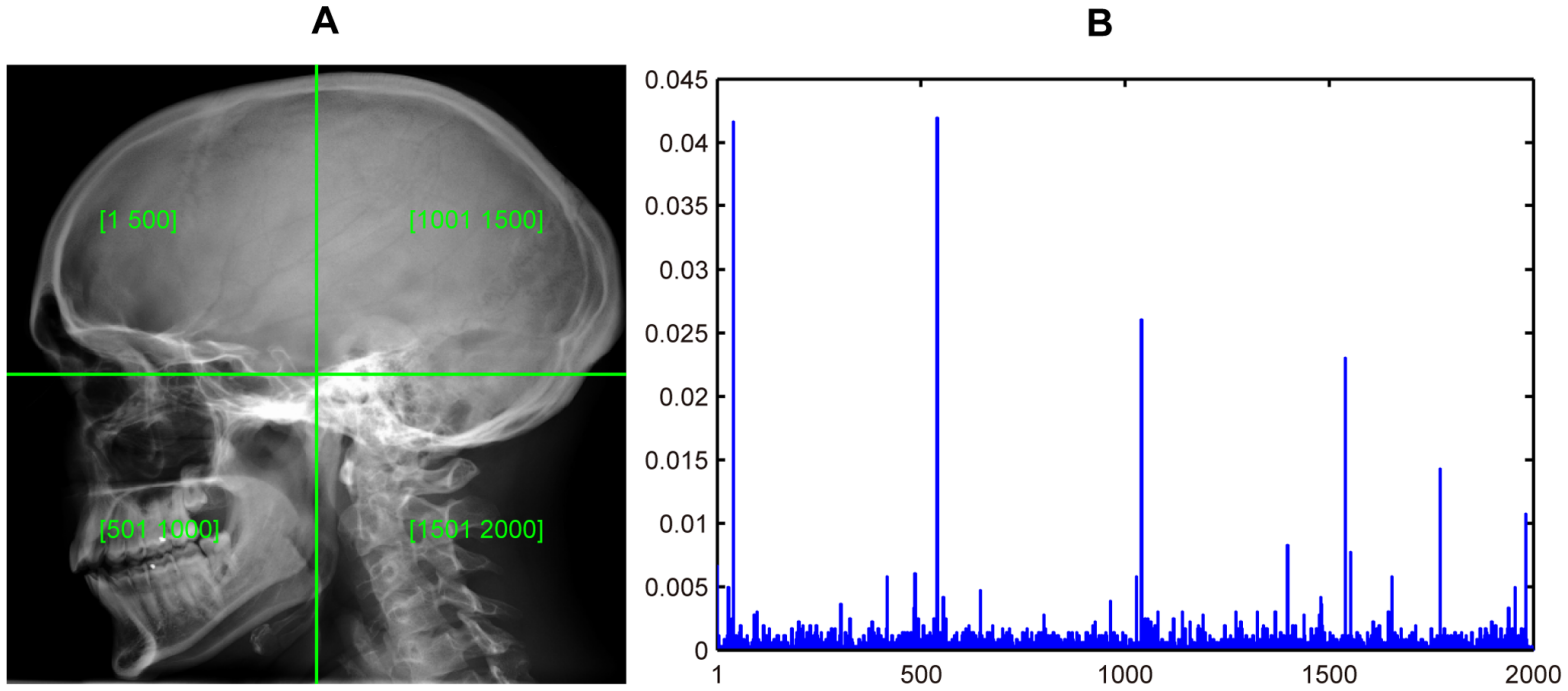
A medical image and its SIFT histogram. (A) Image is equally divided into 2×2 regions. Text presented on each region is the current region SIFT histogram coordinate interval in the concatenated histogram shown in (B). (B) Concatenated SIFT histogram.

To enhance the discriminability of the SIFT descriptor, we also divide each image equally into 1×1, 2×2, 3×3 and 4×4 regions, respectively. From each region, a 500-bin SIFT histogram is generated. By concatenating and normalizing these SIFT histograms, we obtain a long vector to represent the whole image. Thus for each image, we obtain four different SIFT features: 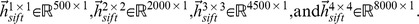 **Figure 3** illustrates a 2×2 division scenario and the corresponding normalized concatenated SIFT histogram.

#### Pixel intensity

The raw intensity value of each image pixel is also utilized as a content descriptor to represent the image. We follow the bag of features paradigm and dense sampling strategy to generate intensity histograms from medical images. The parameter settings of dense sampling and visual word dictionary building are the same as those detailed in Section 3.2.2. We utilize a 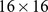 image patch 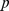 to densely sample each image region. Therefore, we obtain an intensity vector 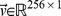 by concatenating the intensity values of 256 pixels contained in 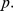 We also utilize K-means clustering to generate an intensity visual word dictionary 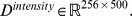 Via histogram accumulation, we finally obtain a 500-bin intensity histogram to represent the sampled image or image region.

**Figure 4:**
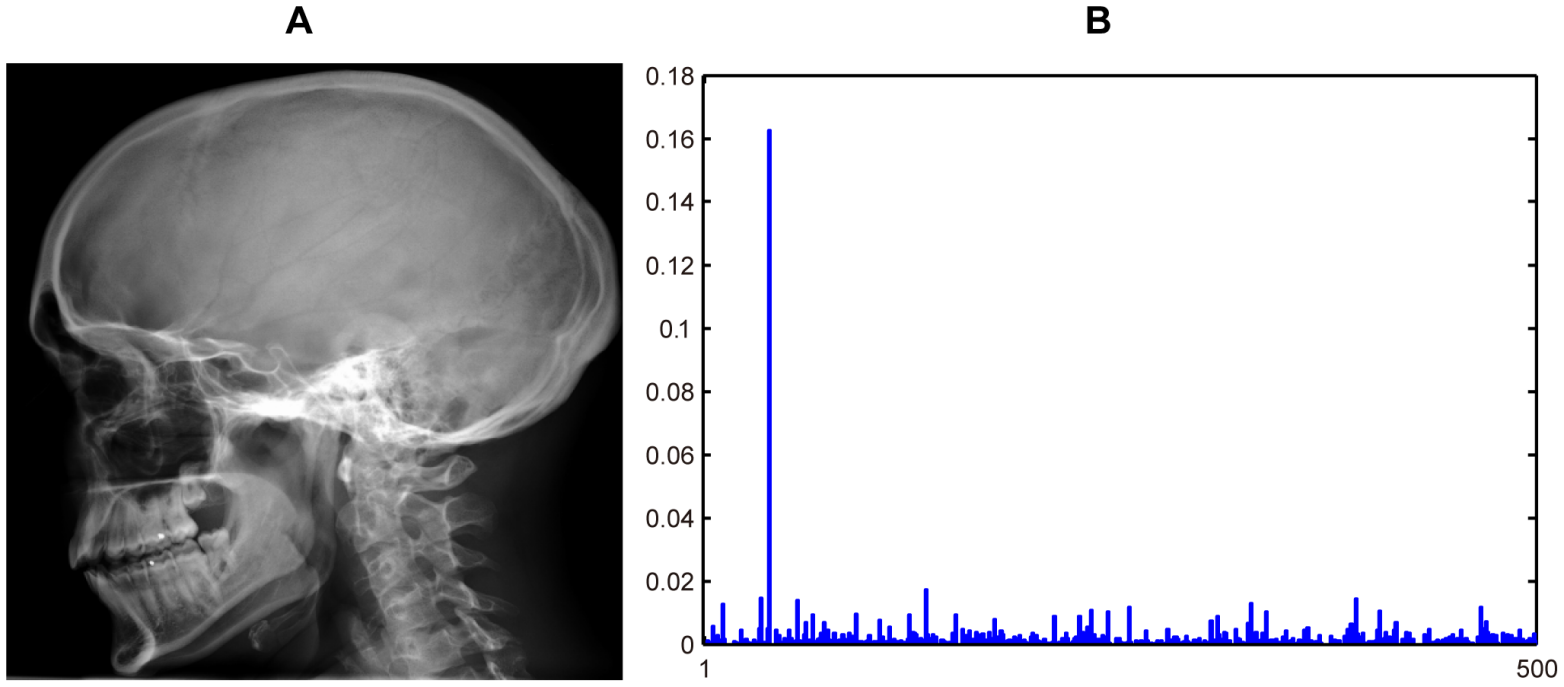
A medical image and its intensity histogram. (A) Original image. (B) Normalized intensity histogram.

To enhance the discriminability of the intensity descriptor, we also divide each image equally into 1×1, 2×2, 3×3 and 4×4 regions, respectively. An intensity histogram is built from each region. Finally, a histogram of the whole image is obtained by concatenating the region intensity histograms into a long vector. Thus for each image, we finally obtain four intensity feature vectors: 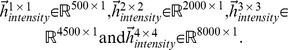 **Figure 4** shows the 1×1 division scenario and the corresponding normalized intensity histogram.

## Results

This section evaluates performance of MLLE compared with that of LLE, MSE [31], LE and PCA, in the context of CBMIR. We organize this section as follows. In Section 4.1, we evaluate performance of these dimensionality reduction methods using mean average precision (MAP). In Section 4.2, we use receiver operating characteristic (ROC) curve analysis to evaluate performance of these methods. Section 4.3 reports evaluation results in terms of sensitivity, specificity, and diagnostic odds ratio (DOR). In Section 4.4, we explore effects of parameters 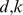 and 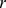 on performance of MLLE. In Section 4.5, we discuss performance discrepancy of MLLE when using different distance metrics to compute the K-nearest neighbors contained in local patch, which is detailed in Section 2.1. In Section 4.6, we conduct experiments to demonstrate that there is no need to perform feature selection before MLLE.

In the following experiments, the subspace dimension 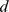 in MLLE, LLE, MSE, PCA and LE is set as 200. The number of nearest neighbors 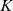 in MLLE, LLE, MSE and LE is fixed to 140. The parameter 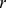 for MLLE, MSE is fixed to 2.5. The procedure for finding optimal parameters 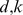 and 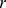 for MLLE is detailed in Section 4.4.

### Performance Evaluations Using MAP

In this section, we use MAP to compare the effectiveness of the proposed MLLE for CBMIR with that of LLE, MSE, PCA and LE.

The experiment is conducted as follows. First, the low-dimensional subspaces of the medical image data set are learned by MLLE, PCA, LLE, MSE and LE, respectively. MLLE simultaneously learns a low-dimensional subspace from twelve features. For the other three methods, low-dimensional subspaces are learned by concatenating all twelve features. Second, based on the learned subspaces, a “leave one out” image retrieval procedure is conducted in the data set. In detail, we choose one image as the query sample for each category; all other images from the data set are ranked according to the Euclidean distance to the query image measured in the low-dimensional subspace. For each query, the top 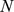 images are returned. In this section, we use MAP to evaluate the performance of a dimension reduction method. MAP is the mean of all average precisions (AP) for different categories. The AP is computed in the ranked top 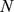 images.

**Figure 5:**
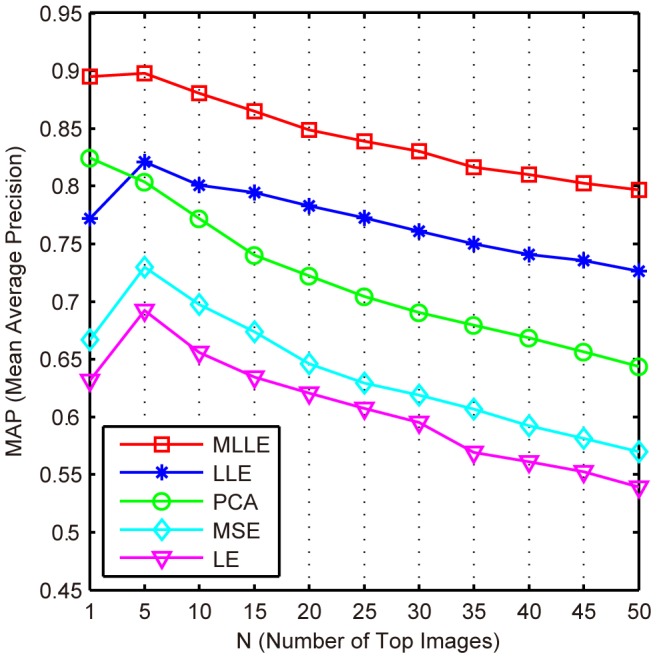
Comparison of the mean average precision of the MLLE, LLE, MSE, PCA and LE methods.

**Figure 5** shows the MAP values when different dimension reduction methods are used. The number of top *N* images starts with one, and increases from five to fifty with step five. The result shows that our MLLE method achieves the best performance. The most effective feature of MLLE is that it benefits from the alternating optimization and global coordinate alignment techniques, which exploit the complementary properties of different features and simultaneously learn a unified low-dimensional subspace from these features.

**Figure 6:**
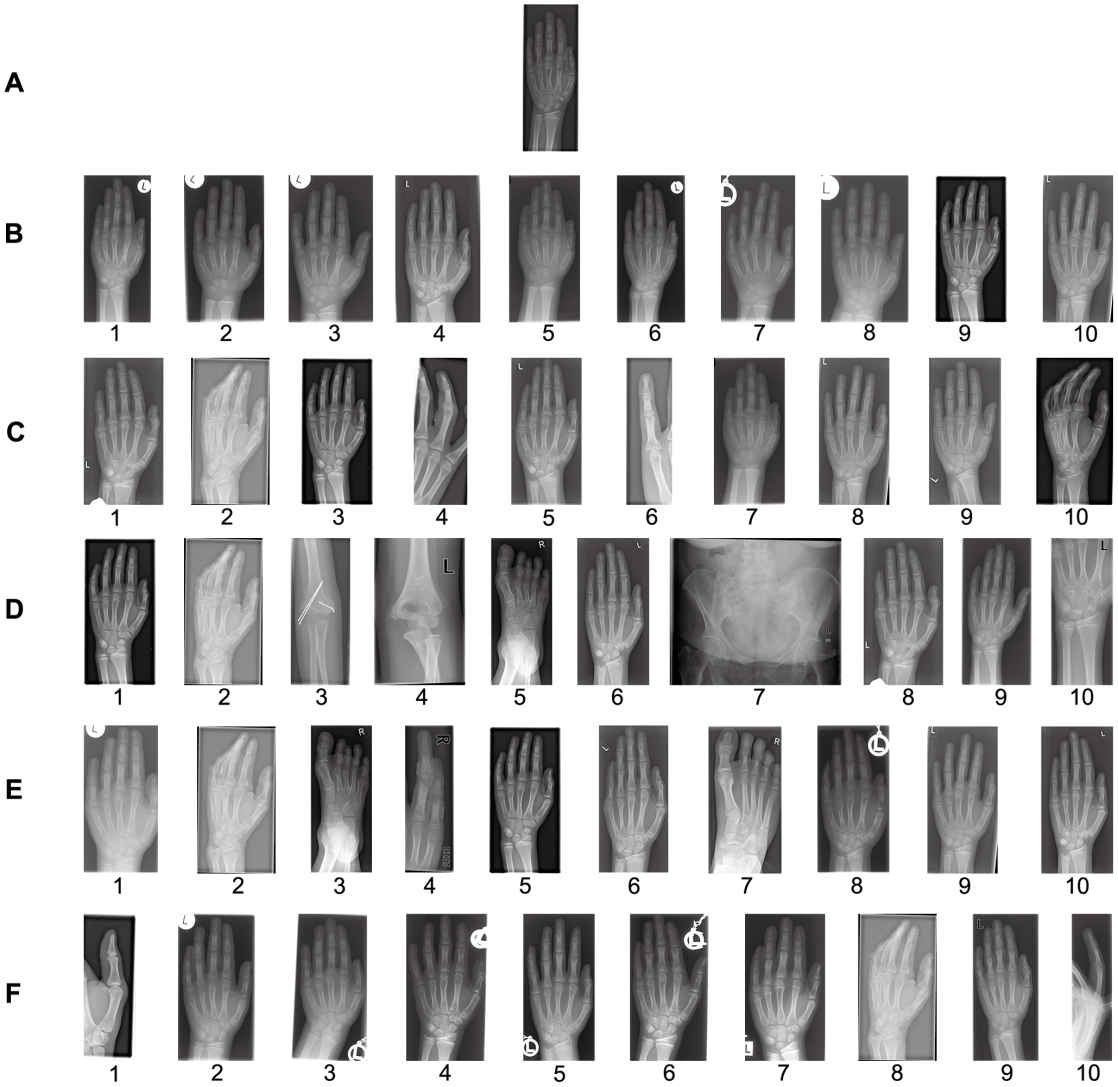
Query and top ten retrieved medical images. (A) Query image. (B) Retrieval results of MLLE. (C) Retrieval results of LLE. (D) Retrieval results of PCA. (E) Retrieval results of LE. (F) Retrieval results of MSE.

To detail the effectiveness of MLLE for CBMIR, we illustrate one of the retrieval results in **Figure 6**. As shown in the figure, there are six rows of medical images. From top to bottom, the first row is the query image, while the other five rows are the retrieval results of MLLE, LLE, PCA, LE and MSE, respectively. Each row of retrieval results consists of the top ten images retrieved from the data set. From the figure, we can see MLLE has the best retrieval performance. In (B), all of the images retrieved by MLLE come from the same category as the query image. In (C), images 2, 4, 6, 10 retrieved by LLE are not similar to the query image. In (D), images 2, 3, 4, 5, 7, 10 are erroneously retrieved by PCA. In (E), images 2, 3, 4, 7 are incorrectly retrieved by LE. Moreover, images 1, 8, 10 in (F) are also erroneously retrieved by MSE.

### Performance Evaluations Using ROC

In this section, we compare performance of MLLE with that of LLE, MSE, LE and PCA using ROC curve analysis.

ROC curve analysis is a popular mechanism to measure the ability of a computer program to determine a given medical image as “positive” or “negative”, which is the typical “two-class” classification problem. And currently, there is no practical methods to assess the performance of “N-class” classification task using ROC curve [45]. We treat CBMIR as a binary classification problem: for a given query image, the task of CBMIR is to classify samples contained in image data set into two classes, i.e., positive class (query image relevant class) and negative class (query image irrelevant class). The IRMA medical image data set used in our experiments contains 57 categories. So we evaluate retrieval performance of MLLE and other dimensionality reduction methods on each IRMA category and plot the corresponding ROC curves, respectively. Because of space limitation, we present here ROC curves obtained on six IRMA categories. ROC curves on other categories can also be obtained with the method detailed as follows.

We conduct two experiments, namely experiment #1 and experiment #2, to perform ROC curve analysis.

**Experiment #1** includes the following two steps. **Step 1:** We project high dimensional medical image samples to 200-dimension subspace using MLLE, LLE, MSE, LE and PCA, respectively. In detail, for MLLE, we simultaneously learn the 200-dimension subspace from 12 visual features. For LLE, MSE, LE and PCA, we first combine 12 visual features into a 31,474-dimension vector. Then we utilize these methods to project the high dimensional dataset to 200 dimensional samples. **Step 2:** We employ binary support vector machines (SVM) as classifier to determine the probability that a given image is positive, based on the learned dimensionality reduced data set. In detail, we use LIBSVM [46] to solve the binary SVM classifier. For each IRMA category, five-fold cross-validation scheme [47] is employed to train the binary SVM classifier. Then we treat all images within current IRMA category as positive test examples for ROC curve analysis. Meanwhile, we utilize images within other categories as negative test examples.

**Experiment #2** also has two steps. This experiment only differs from experiment #1 that k-nearest neighbors (KNN) is used as classifier in step 2. In detail, for a given test sample 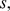 a “leave one out” retrieval is performed. All other images contained in the data set are sorted according to their Euclidean distance to 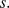 The probability that 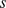 is positive is defined as 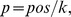 where 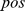 is the number of positive samples among k nearest neighbors of 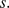 In our experiment, we set 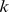 as 15.

**Figure 7:**
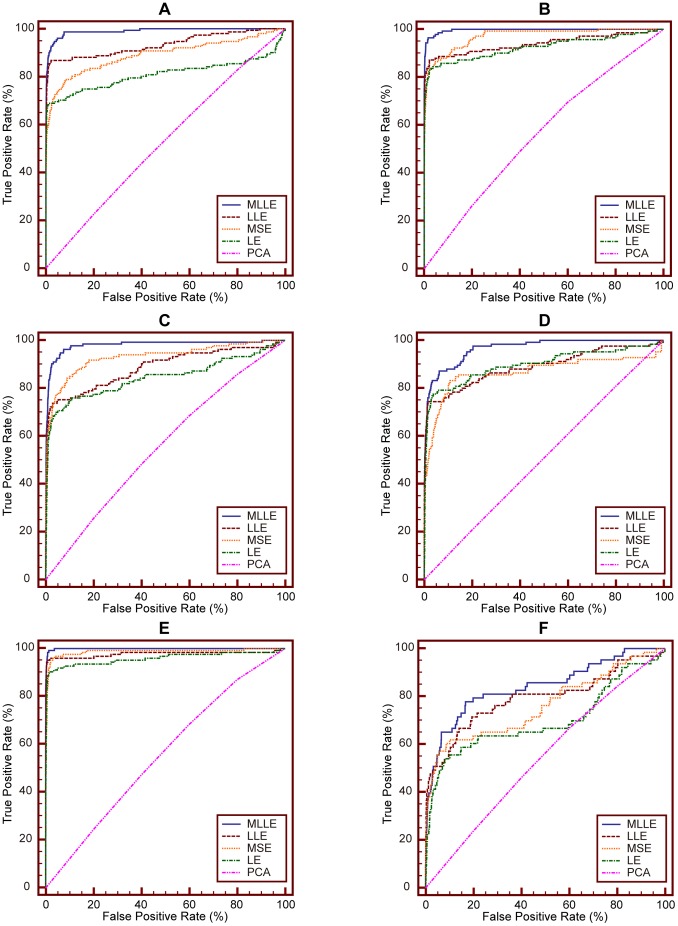
Comparison of ROC curves for MLLE, LLE, MSE, LE and PCA on different IRMA category. The classifier is SVM. (A) ROC curves obtained on IRMA category 14. (B) ROC curves obtained on IRMA category 16. (C) ROC curves obtained on IRMA category 20. (D) ROC curves obtained on IRMA category 21. (E) ROC curves obtained on IRMA category 22. (F) ROC curves obtained on IRMA category 49.

**Figure 8:**
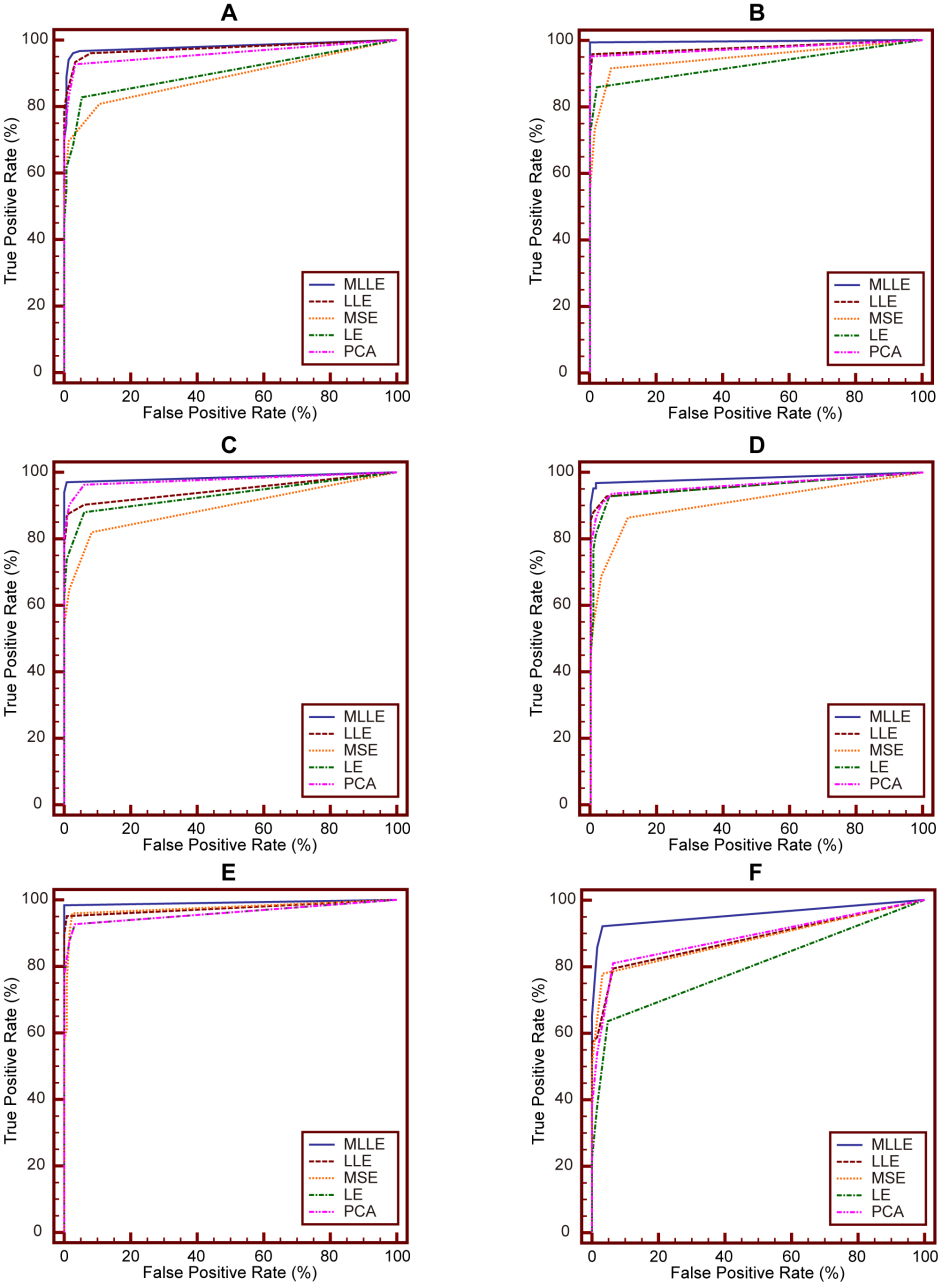
Comparison of ROC curves for MLLE, LLE, MSE, LE and PCA on different IRMA category. The classifier is KNN. (A) ROC curves obtained on IRMA category 14. (B) ROC curves obtained on IRMA category 16. (C) ROC curves obtained on IRMA category 20. (D) ROC curves obtained on IRMA category 21. (E) ROC curves obtained on IRMA category 22. (F) ROC curves obtained on IRMA category 49.

We conduct ROC curve analysis on the IRMA category 14, 16, 20, 21, 22 and 49, respectively. For each IRMA category, number of samples contained in positive and negative test set is detailed in **Table 2**. **Figure 7** shows ROC curves obtained via **experiment #1**. In the experiment, we use SVM as classifier. **Table 3** details the corresponding area under ROC curve (A_Z_ value). **Figure 8** presents ROC curves obtained via experiment #2. In the experiment, we use KNN (K = 15) as classifier. **Table 4** reports the corresponding A_Z_ value. These results are obtained using statistical software MedCalc® 12.7.0.

**Table 2:** Number of samples contained in positive/negative test set used for performance evaluation of different dimensionality reduction methods on different IRMA category.

IRMAcategory	14	16	20	21	22	49
Positivetest set	151	141	133	125	123	63
Negativetest set	10,751	10,761	10,769	10,777	10,779	10,839

**Table 3:** A_Z_ values of different dimensionality reduction methods on different IRMA category.

IRMAcategory	14	16	20	21	22	49
MLLE	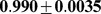	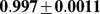	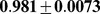	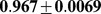	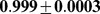	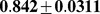
LLE	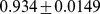	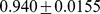	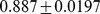	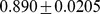	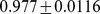	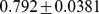
MSE	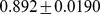	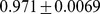	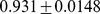	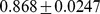	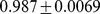	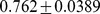
LE	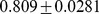	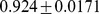	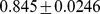	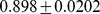	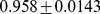	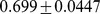
PCA	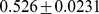	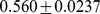	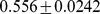	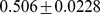	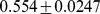	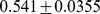

The classifier is SVM.

**Table 4:** A_Z_ values of different dimensionality reduction methods on different IRMA category.

IRMAcategory	14	16	20	21	22	49
MLLE	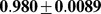	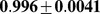	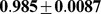	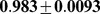	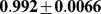	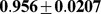
LLE	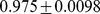	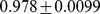	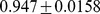	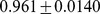	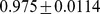	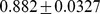
MSE	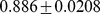	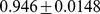	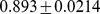	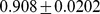	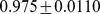	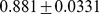
LE	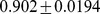	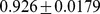	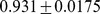	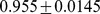	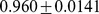	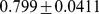
PCA	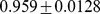	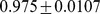	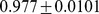	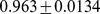	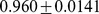	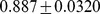

The classifier is KNN.

From **Table 3** we can see that the A_Z_ value for determining between 151 positive images from IRMA category 14 and 10,751 negative images from other categories is 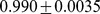 when using the proposed MLLE. When applying LLE, MSE, LE and PCA to distinguish positive and negative images, the computed A_Z_ values are 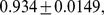 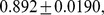 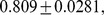 and 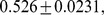 respectively. **Figure 7** (A) represents the comparison of ROC curves for these five sets of performance data. **Table 3** demonstrates that MLLE yields the highest A_Z_ value in discrimination of IRMA category 14 compared to LLE, MSE, LE and PCA ( 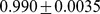 vs. 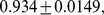 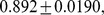 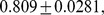 and 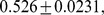 respectively).

The computed A_Z_ values for detecting between positive and negative images from IRMA category 16, 20, 21, 22 and 49 are also detailed in **Table 3**. The corresponding comparison of ROC curves is demonstrated in **Figure 7** (B), (C), (D), (E), and (F), respectively. The results indicate that MLLE achieves best performance than traditional dimensionality reduction methods. We can draw the same conclusion by analyzing **Figure 8** and **Table 4**.

Another phenomenon should be noted is the significant performance difference of PCA between experiment #1 and experiment #2. From **Figure 7** and **Table 3**, we can see that PCA achieves poor performance (A_Z_ value of PCA on IRMA category 14, 16, 20, 21, 22 and 49 is 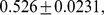 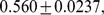 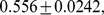 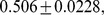 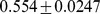 and 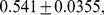 respectively). Moreover, the performance of PCA is worse than that of other methods. While **Figure 8** and **Table 4** demonstrate that PCA gains significant performance improvement (A_Z_ value of PCA on IRMA category 14, 16, 20, 21, 22 and 49 is 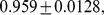 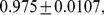 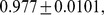 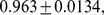 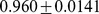 and 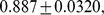 respectively). And the performance of PCA is better than that of MSE and LE.

Based on these two experiments, we conclude that PCA performs poorly in experiment #1 is caused by the subsequent classifier, SVM. We further discuss the reason as follows.

PCA maximizes the mutual information between original high dimensional Gaussian distributed samples and projected low-dimensional samples. It does not explore the geometric structure of the data. Therefore, in the very low dimensional subspace projected by PCA, when there exists great imbalance between positive and negative set (as shown in **Table 2**), it is hard for SVM to find the optimal hyperplane to separate positive set from negative set.

Different to PCA, MLLE, LLE, MSE and LE are manifold learning based dimensionality reduction methods. These methods explore geometric structure among samples in high dimensional data set, and preserve the structure in low dimensional sub-space. Therefore, though great imbalance exists between positive and negative set, it is possible for SVM to find the optimal hyperplane to separate positive set from negative set. Because geometric structure of positive and negative set is preserved in the low dimensional data set, respectively. Then performance of MLLE, LLE, MSE and LE does not greatly affected by classifiers. We can draw the conclusion from **Table 3** and **Table 4**.

### Performance Evaluations Using Sensitivity, Specificity, and DOR

In this section, we compare performance of MLLE with that of LLE, MSE, LE and PCA using sensitivity, specificity, and DOR.

Sensitivity, specificity and DOR are indicators to compare performance of competing diagnostic tests, which are used to separate subjects with a target disorder from subjects without it [48]. Diagnostic test is the typical “two-class” classification problem: for a given subject, the aim of diagnostic test is to determine whether the subject is “positive” (with a target disorder) or “negative” (without a target disorder).

Following this, we design experiments to evaluate diagnostic performance of MLLE, LLE, MSE, LE and PCA on each category of IRMA data set, respectively. In detail, for each IRMA category, we treat it as positive test set. Meanwhile, a negative test set containing equal number of samples as that of positive test set is constructed by randomly selecting images from other categories. Based on the positive test set and negative test set, a diagnostic test procedure is performed on low-dimensional embedding obtained by MLLE, LLE, MSE, LE and PCA, respectively. Definitely, for each test image, all other images contained in IRMA data set are ranked according to their L2 distances to the test image. Then diagnostic result of the test image is determined by the following criterion: if more than half of the top k ranked images is positive, then the test image is positive; otherwise, the test image is negative. In our experiments, we set k as 15.

Similar to ROC curve analysis, we present here experimental results obtained on four IRMA categories. Experimental results on other categories can also be obtained with the method detailed above.

**Table 5**, **Table 6**, **Table 7** and **Table 8** compare diagnostic performance of MLLE, LLE, MSE, LE and PCA in terms of sensitivity, specificity and DOR, which are obtained on IRMA category 1, 4, 7 and 25, respectively. We get these results using Meta-Disc 1.4 [49]. As shown in **Table 5**, the estimated sensitivity, specificity and DOR for the proposed MLLE in determining images from category 1 is 0.92 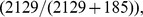 0.99 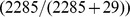 and 906.76 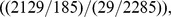 respectively. This means that for MLLE the odds for positivity among medical images from IRMA category 1 are 906.76 times higher than the odds for positivity among medical images from other IRMA categories. In the same way, the DORs for LLE, MSE, LE and PCA can be calculated. From **Table 5** we can draw the conclusion that MLLE has the highest DOR in discrimination of IRMA category 1 compared to LLE, MSE, LE and PCA (906.76 vs. 773.44, 523.27, 335.32 and 675.00, respectively). The same conclusion can be drawn from **Table 6**, **Table 7** and **Table 8**.

**Table 5:** Comparison of sensitivity, specificity and DOR for MLLE, LLE, MSE, LE and PCA on IRMA category 1.

Methods	Sensitivity		Specificity		DOR		TP	FP	FN	TN
	%	(95% CI)	%	(95% CI)		(95% CI)				
MLLE	**92**	**91−93**	**99**	**98−99**	**906.76**	**610.33−1,347.15**	2,129	29	185	2,285
LLE	92	91−93	98	98−99	773.44	537.72−1,112.49	2,139	36	175	2,278
MSE	88	87−90	99	98−99	523.27	362.77−754.79	2,044	33	270	2,281
LE	87	86−89	98	97−99	335.32	244.98−458.96	2,023	47	291	2,267
PCA	89	87−90	99	98−99	675.00	452.06−1,007.89	2,056	27	258	2,287

**Table 6:** Comparison of sensitivity, specificity and DOR for MLLE, LLE, MSE, LE and PCA on IRMA category 4.

Methods	Sensitivity		Specificity		DOR		TP	FP	FN	TN
	%	(95% CI)	%	(95% CI)		(95% CI)				
MLLE	**97**	**95−99**	**100**	**99−100**	**14,909.09**	**1,915.96−116,015.2**	400	1	11	410
LLE	92	89−94	100	98−100	2,342.45	558.26−9,828.84	378	2	33	409
MSE	67	62−72	99	97−100	166.01	67.12−410.61	276	5	135	406
LE	84	80−88	99	97−100	432.23	172.11−1,085.52	346	5	65	406
PCA	93	90−95	99	97−99	857.25	352.87−2,082.55	381	6	30	405

**Table 7:** Comparison of sensitivity, specificity and DOR for MLLE, LLE, MSE, LE and PCA on IRMA category 7.

Methods	Sensitivity		Specificity		DOR		TP	FP	FN	TN
	%	(95% CI)	%	(95% CI)		(95% CI)				
MLLE	**96**	**93−98**	**99**	**97−100**	**2,158.60**	**587.19−7,935.28**	251	3	10	258
LLE	92	88−95	99	97−100	982.86	289.47−3,337.20	240	3	21	258
MSE	56	50−62	99	97−100	166.99	40.66−685.74	147	2	114	259
LE	79	74−84	99	97−100	496.42	119.62−2,060.15	207	2	54	259
PCA	90	85−93	99	97−100	1,122.33	264.02−4,771.06	234	2	27	259

**Table 8:** Comparison of sensitivity, specificity and DOR for MLLE, LLE, MSE, LE and PCA on IRMA category 25.

Methods	Sensitivity		Specificity		DOR		TP	FP	FN	TN
	%	(95% CI)	%	(95% CI)		(95% CI)				
MLLE	**83**	**74−89**	**99**	**95−100**	**522.05**	**68.56−3,975.17**	91	1	19	109
LLE	66	57−75	100	97−100	433.16	26.19−7,164.50	73	0	37	110
MSE	39	30−49	99	95−100	69.96	9.41−519.93	43	1	67	109
LE	34	25−43	100	97−100	112.76	112.76−1,864.98	37	0	73	110
PCA	57	47−67	100	97−100	295.44	295.44−4,875.03	63	0	47	110

Evaluation results in terms of sensitivity, specificity, and DOR show that the proposed MLLE yields significantly higher performance than traditional dimensionality reduction methods.

### Effects of Parameters

In this section, we analyze effects of parameters on MLLE performance. These parameters include 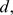 dimension of the learned embedding, 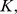 number of nearest neighbors contained in local patch, and 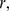 scaling factor for the weight of each feature.

#### Effects of parameter *d*

**Figure 9:**
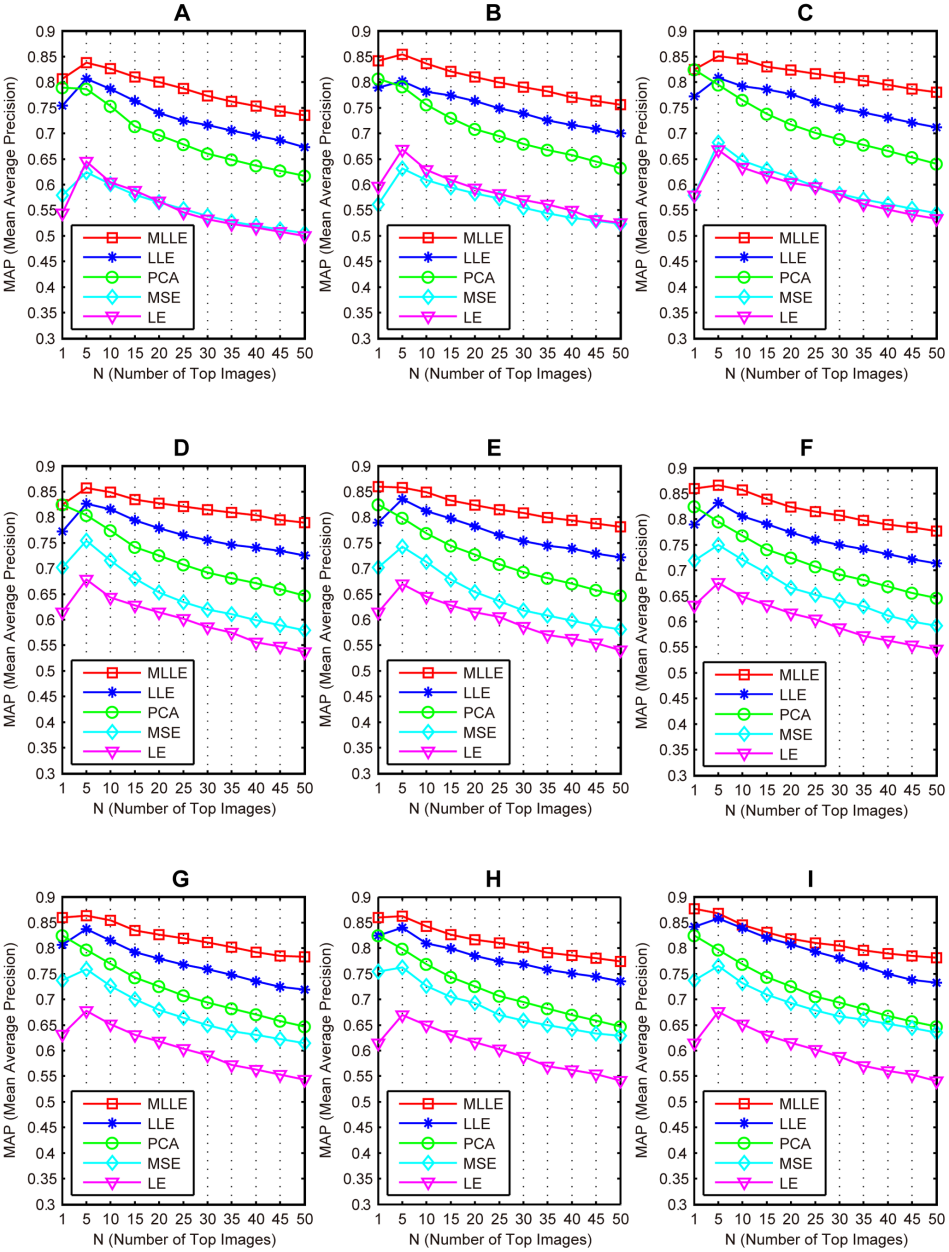
Performance of proposed MLLE compared with existing methods. (A) The algorithms are evaluated with 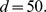 (B) The algorithms are evaluated with 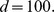 (C) The algorithms are evaluated with 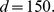 (D) The algorithms are evaluated with 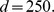 (E) The algorithms are evaluated with 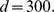 (F) The algorithms are evaluated with 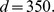 (G) The algorithms are evaluated with 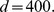 (H) The algorithms are evaluated with 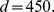 (I) The algorithms are evaluated with 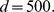

Figure 9 shows the MAP values when the propose MLLE is evaluated using different dimensionalities 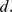 In these experiments, parameters 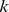 and 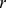 are same as those in the former experiment. From these experiments, we can see that the proposed MLLE outperforms existing dimension reduction methods. Moreover, we detail the MAP values of MLLE in Table 9. From the table we can see that MLLE achieves the best performance with 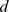 set as 200.

**Table 9:** Mean average precision values of MLLE evaluated with different *d.*

TOP N	N = 1	N = 5	N = 10	N = 15	N = 20	N = 25	N = 30	N = 35	N = 40	N = 45	N = 50
*d* = 50	0.8070	0.8381	0.8265	0.8101	0.7999	0.7874	0.7729	0.7626	0.7537	0.7433	0.7349
*d* = 100	0.8421	0.8541	0.8365	0.8210	0.8098	0.7996	0.7908	0.7818	0.7703	0.7631	0.7563
*d* = 150	0.8246	0.8507	0.8454	0.8303	0.8236	0.8166	0.8093	0.8026	0.7948	0.7867	0.7809
***d*** ** = 200**	**0.8947**	**0.8980**	**0.8807**	**0.8650**	**0.8488**	**0.8391**	**0.8302**	**0.8163**	**0.8102**	**0.8026**	**0.7967**
*d* = 250	0.8246	0.8575	0.8494	0.8344	0.8269	0.8211	0.8151	0.8097	0.8037	0.7946	0.7891
*d* = 300	0.8596	0.8581	0.8491	0.8326	0.8241	0.8150	0.8086	0.7993	0.7941	0.7877	0.7816
*d* = 350	0.8596	0.8660	0.8568	0.8389	0.8237	0.8151	0.8073	0.7976	0.7896	0.7840	0.7767
*d* = 400	0.8596	0.8636	0.8543	0.8348	0.8265	0.8194	0.8108	0.8022	0.7926	0.7852	0.7829
*d* = 450	0.8596	0.8625	0.8431	0.8268	0.8166	0.8098	0.8018	0.7914	0.7855	0.7804	0.7737
*d* = 500	0.8772	0.8680	0.8455	0.8309	0.8182	0.8099	0.8051	0.7955	0.7893	0.7846	0.7816

#### Effects of parameter *K*

**Figure 10:**
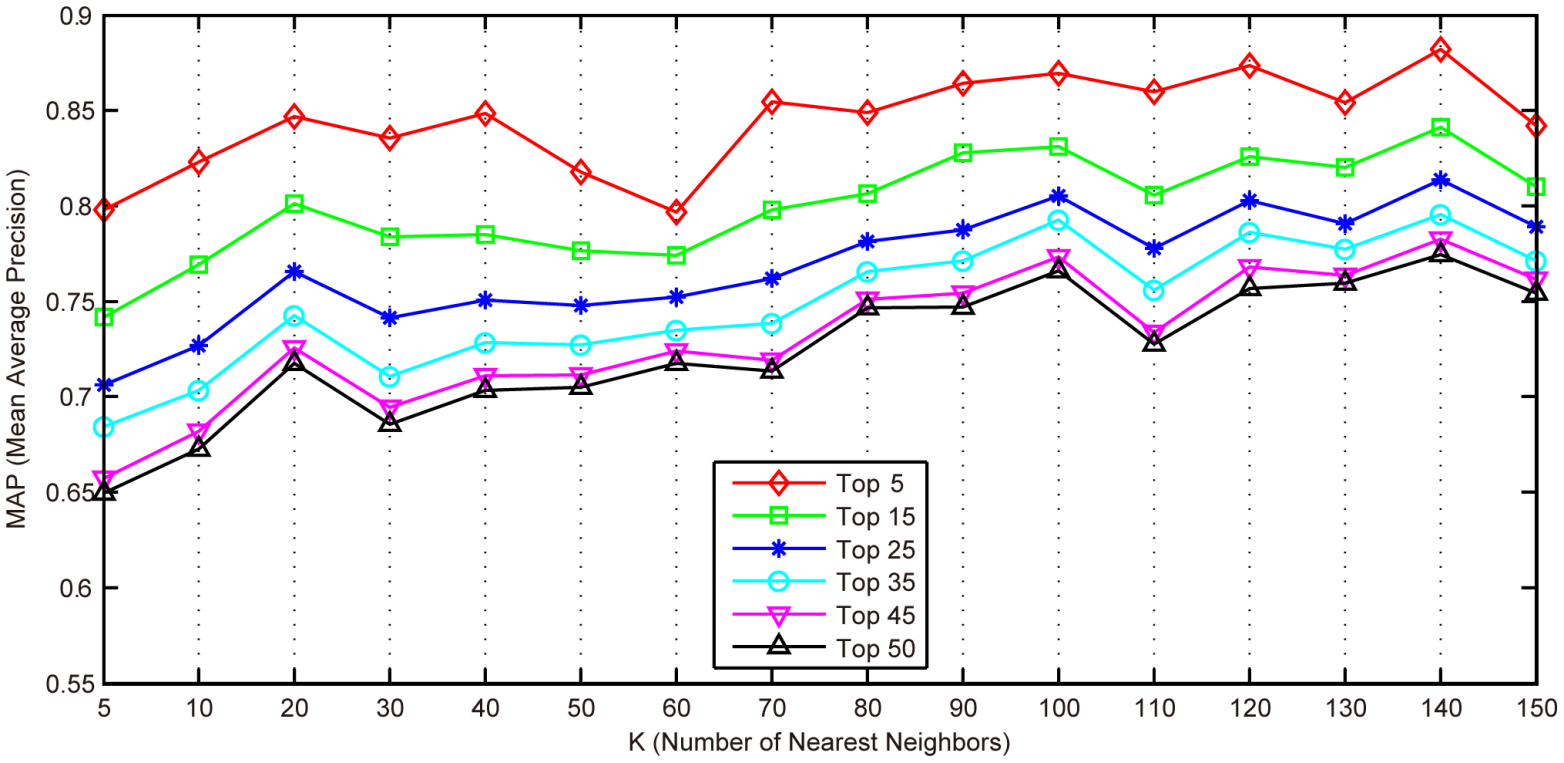
Mean average precision values of the proposed MLLE evaluated with different 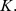

Figure 10 shows the MAP values when the proposed MLLE is evaluated with different 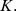 In the experiments, parameters 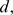 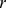 are fixed to 200 and 2, respectively. The results show that MLLE achieves the best performance with 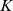 set as 140.

#### Effects of parameter *r*

**Figure 11:**
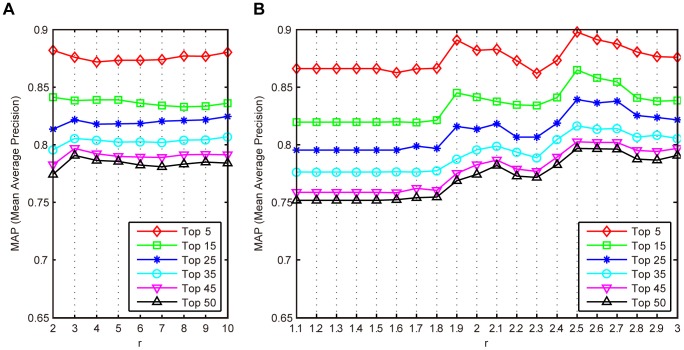
Mean average precision of the proposed MLLE evaluated with different 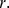 (A) 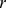 is updated from 2 to 10 with step 1. (B) 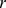 is updated from 1.1 to 3 with step 0.1.

**Figure 11** shows the MAP values when MLLE is evaluated with different 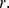 In the experiments, parameters 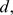 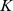 are fixed to 200 and 140, respectively. In **Figure 11** (A), 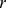 is updated from 2 to 10 with step 1. From the figure, we can see that MLLE achieves best performance when 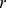 is approximate to 3. In **Figure 11** (B), 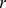 is updated from 1.1 to 3 with step 0.1. It can be seen that MLLE achieves best performance when 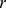 is set as 2.5.

### Performance Comparison of MLLE with Different Distance Metrics

Geodesic distance, L1 distance (which is also named city block distance or Manhattan distance) and L2 distance are well-known distance metrics used in the field of dimensionality reduction. In Section 2.1, we use L2 distance to find K-nearest neighbors of each medical image. In this section, we perform experiments to evaluate performance of MLLE with different distance metrics, i.e., geodesic, L1, and L2 distance.

Following the same experiment setup of experiment #1 detailed in Section 4.2, we conduct experiments to evaluate effects of these three different distance metrics on MLLE performance using ROC curve analysis.

**Figure 12:**
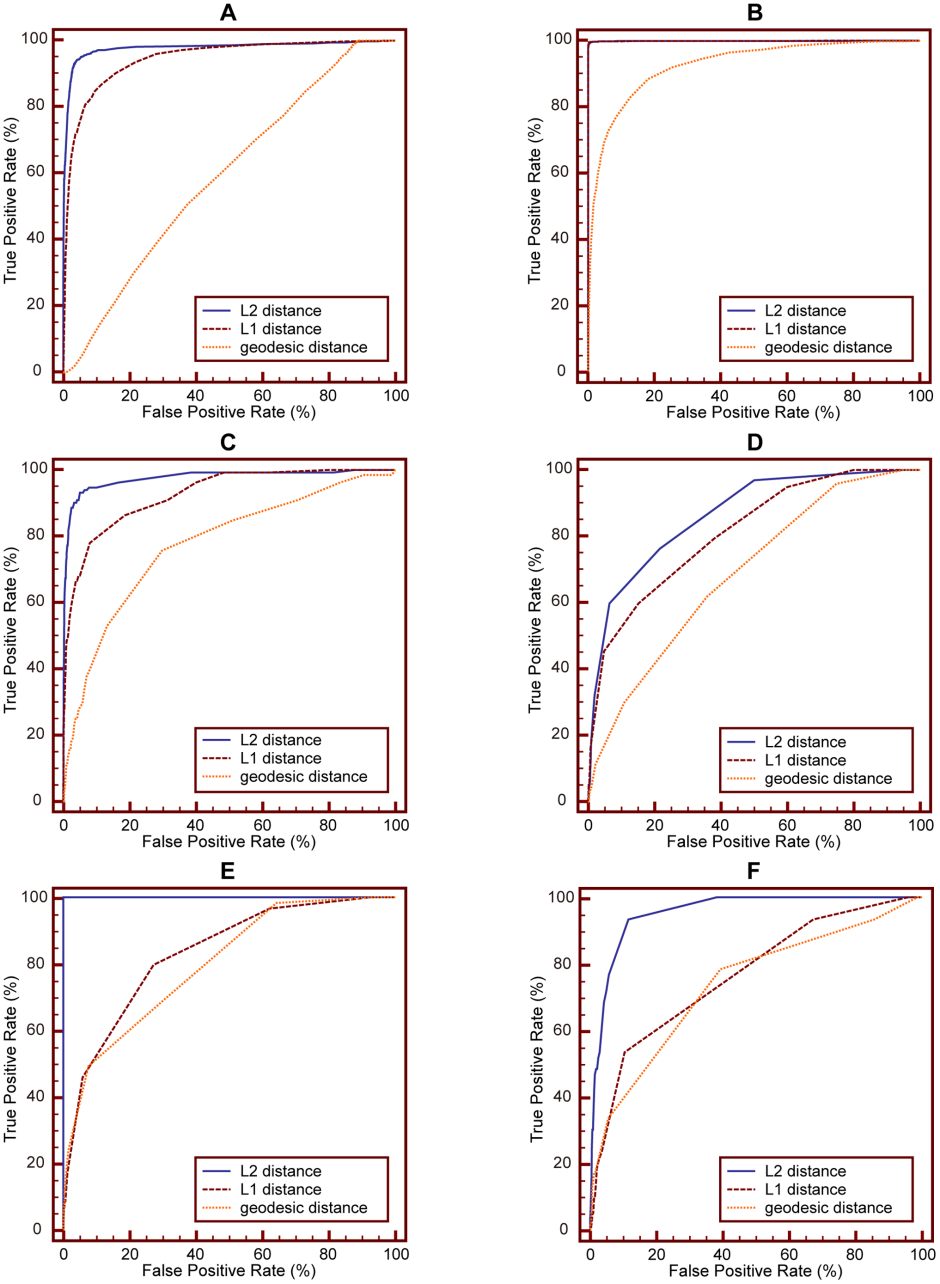
Comparison of ROC curves for MLLE with L2, L1, and geodesic distance on different IRMA category. (A) ROC curves obtained on IRMA category 2. (B) ROC curves obtained on IRMA category 3. (C) ROC curves obtained on IRMA category 19. (D) ROC curves obtained on IRMA category 31. (E) ROC curves obtained on IRMA category 51. (F) ROC curves obtained on IRMA category 52.

**Figure 12** shows ROC curves of MLLE with different distance metrics obtained on IRMA category 2, 3, 19, 31, 51 and 52, respectively. The number of images contained in positive and negative test set for each category is presented in **Table 10**. **Table 11** details the corresponding A_Z_ values.

**Table 10:** Number of samples contained in positive/negative test set used for performance evaluation of MLLE with different distance metrics on different IRMA category.

IRMA category	2	3	19	31	51	52
Positive test set	1,103	1,042	132	97	59	60
Negative test set	9,799	9,860	10,770	10,805	10,843	10,842

**Table 11:** A_Z_ values of MLLE with different distance metrics on different IRMA category.

IRMAcategory	2	3	19	31	51	52
L2 distance	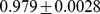	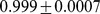	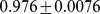	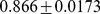	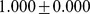	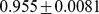
L1 distance	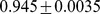	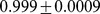	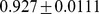	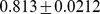	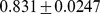	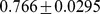
geodesic distance	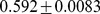	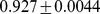	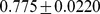	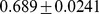	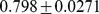	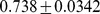

As shown in **Table 11**, for IRMA category 2, the A_Z_ value for detecting between 1,103 positive images and 9,799 negative images is 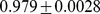 when using L2 distance. When applying L1 distance and geodesic distance, the computed A_Z_ values are 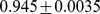 and 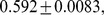 respectively. **Figure 12** (A) shows the comparison of ROC curves for these three sets of performance data. **Table 11** demonstrates that L2 distance achieves the highest A_Z_ value in detection of IRMA category 2 compared to L1 distance and geodesic distance ( 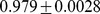 vs. 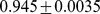 and 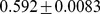, respectively).

The computed A_Z_ values for detecting between positive and negative images from IRMA category 3, 19, 31, 51 and 52 are also detailed in **Table 11**. The corresponding ROC curves are demonstrated in **Figure 12** (B), (C), (D), (E) and (F), respectively. From these results we can conclude that L2 distance is the best solution for MLLE to construct local patches. The same conclusion can be drawn from experimental results obtained on other IRMA categories.

### Selecting Features before MLLE

In this section, we conduct experiments to demonstrate that there is no need to perform feature selection before MLLE.

The proposed MLLE has the merit of simultaneously learning a low-dimensional embedding from multiple features, by exploring different significances of different features. In detail, MLLE assumes that each feature has different contribution to the final learned low-dimensional embedding, though the feature does not have significant difference between different medical images. We clarify this point based on two experiments described as follows.

**Experiment #3** includes the following three steps. **Step 1**: For each medical image 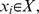 we divide its twelve features into three groups: LBP group 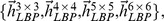 SIFT group 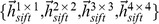 and intensity group 
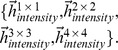
 **Step 2**: For each group, we employ *laplacian score feature selection (LPFS)* [50], the unsupervised feature selection method, to determine the importance of each feature. In detail, within each feature group, we concatenate the four feature vectors into a long vector. So we get three long feature vectors to represent 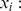 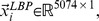 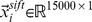 and 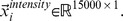 Then the medical image data set 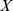 has three different views: 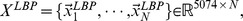 
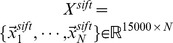
 and 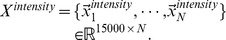 On each view, we use LPFS to determine the importance of each feature. And the most important 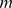 feature entries are selected. Finally, 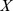 is represented by three dimension-reduced views: 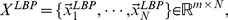 
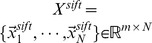
 and 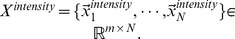 Accordingly, for each image 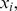 we obtain three dimension-reduced feature vectors: 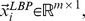 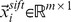 and 
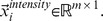
 In our experiment, we set 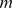 as 500. **Step 3**: We utilize MLLE to learn the low-dimensional embedding 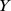 based on three views obtained in step 2. The dimension of 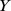 is set as 200. We denote this method as lpfs-MLLE (laplacian score feature selection-based MLLE).

**Experiment #4** includes the following three steps. **Step 1**: This step is same as step 1 of experiment #3. **Step 2**: For each feature group, we employ *multi-cluster feature selection (MCFS)* [51], the manifold learning-based feature selection method, to select features which can best preserve the multi-cluster structure of medical image data set 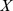. In detail, each medical image 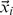 has three different feature vectors: 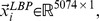 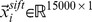 and 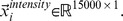 Then the whole medical image data set 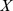 can be represent by three different views: 
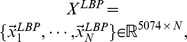
 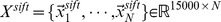 and 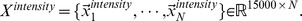 On each view, we use MCFS to select 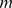 feature entries which can best preserve the multi-class structure of this view. In our experiment, we set 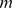 as 500. Then 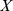 can be represented by three dimension-reduced views: 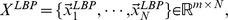 
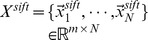
 and 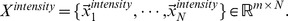 **Step 3**: This step is same as step 3 of experiment #3. We denote this method as mcfs-MLLE (Multi-cluster feature selection-based MLLE).

**Figure 13:**
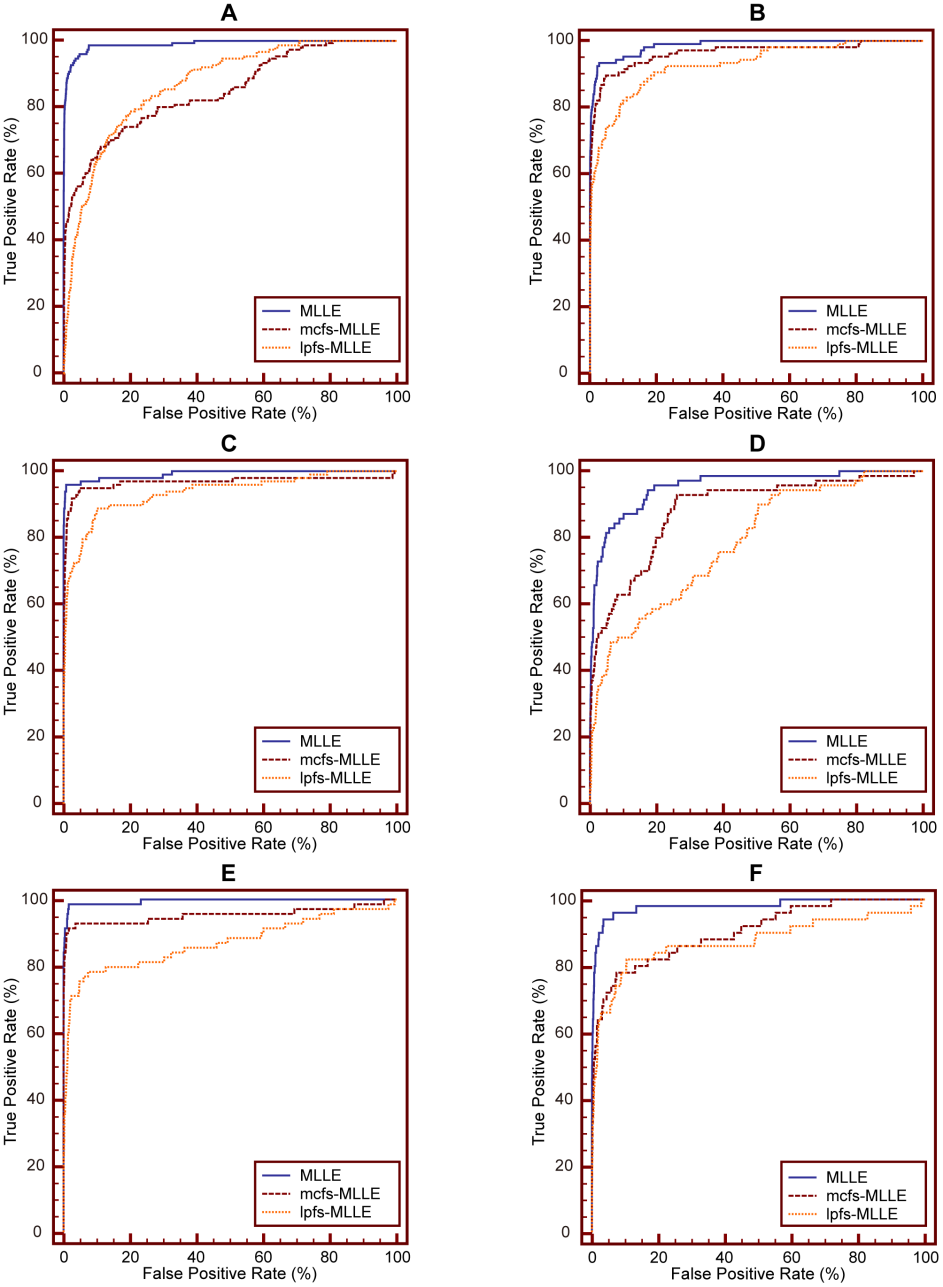
Comparison of ROC curves for MLLE, mcfs-MLLE and lpfs-MLLE on different IRMA category. (A) ROC curves on IRMA category 14. (B) ROC curves on IRMA category 27. (C) ROC curves on IRMA category 30. (D) ROC curves on IRMA category 43. (E) ROC curves on IRMA category 45. (F) ROC curves on IRMA category 57.

We compare performance of MLLE, mcfs-MLLE and lpfs-MLLE using ROC curve analysis. The experimental setup is same as that of experiment #1 detailed in subsection 4.2. **Figure 13** shows ROC curves of these methods obtained on IRMA category 14, 27, 30, 43, 45 and 57, respectively. For each category, the number of samples contained in positive test set and negative test set is detailed in **Table 12**. **Table 13** shows the corresponding A_Z_ values.

**Table 12:** Number of samples contained in positive/negative test set used for performance evaluation of MLLE, mcfs-MLLE and lpfs-MLLE on different IRMA category.

IRMA category	14	27	30	43	45	57
Positive test set	151	106	98	70	69	50
Negative test set	10,751	10,796	10,804	10,832	10,833	10,852

**Table 13:** A_Z_ values of MLLE, mcfs-MLLE and lpfs-MLLE on different IRMA category.

IRMAcategory	14	27	30	43	45	57
MLLE	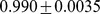	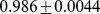	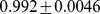	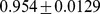	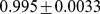	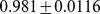
mcfs-MLLE	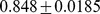	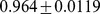	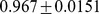	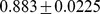	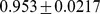	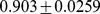
lpfs-MLLE	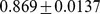	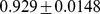	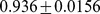	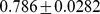	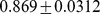	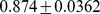

**Table 13** shows that the A_Z_ value for discriminating between 151 positive images from IRMA category 14 and 10,751 negative images from other categories is 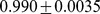 when using MLLE without feature selection. When applying MCFS and LPFS before MLLE to perform the same experiment, the computed A_Z_ values are 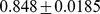 and 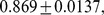 respectively. **Figure 13** (A) demonstrates the comparison of ROC curves for these three sets of performance data. From **Table 13** we can see that directly using MLLE to perform dimensionality reduction yields the highest A_Z_ value in the discrimination of IRMA category 14, compared to using feature selection methods MCFS and LPFS before conducting MLLE ( 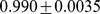 vs. 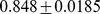 and 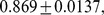 respectively).

The computed A_Z_ values for detecting positive and negative images from IRMA category 27, 30, 43, 45 and 57 are also detailed in **Table 13**. The corresponding comparison of ROC curves are shown in **Figure 13** (B), (C), (D), (E) and (F), respectively. Based on these results, we can come to the conclusion that, though using dimensionality reduction methods before MLLE can reduce features and save computing time, the learned embedding is worse than that obtained directly by MLLE.

It should be noted that, in this manuscript, to demonstrate the effectiveness of MLLE to explore complementary properties of different features, we extract twelve different features from each medical image. In practice, there is a trade-off between the number of visual features and retrieval performance. Within an acceptable range of retrieval performance, users can extract less visual features to save computing time. In fact, three to six visual features are capable of achieving the acceptable retrieval performance.

## Discussion and Conclusion

We organize this section as follows. In Section 5.1, we give statistical analysis of experimental results presented above. Then we discuss the reason that MLLE achieves effective performance than existing dimensionality reduction methods in Section 5.2. Finally, Section 5.3 concludes our work.

### Statistical Analysis

In this paper, we use MAP, DOR and ROC as criteria to evaluate the performance of different methods. These criteria reflect the effectiveness of these methods from different aspects. In particular, MAP demonstrates the retrieval performance of different methods on the IRMA test set. DOR and ROC show the ability of different methods to distinguish different types of medical image. Evaluation results obtained from different criteria demonstrate that MLLE achieves best results.

Statistically, we utilize F1-measure to determine the reliability of different criterion. **Table 14** shows F1-measure values for MLLE, LLE, MSE, LE and PCA on the IRMA category 1, 4, 7 and 25, respectively. From the table, we can see that MLLE achieves the best performance compared with other methods. This evaluation further confirms the results obtained by DOR. By using F1-measure to other performance criteria, i.e., MAP and ROC, we can obtain the same conclusion.

**Table 14:** Comparison of F1-measure values for MLLE, LLE, MSE, LE and PCA on the IRMA category 1, 4, 7 and 25.

IRMA categoryMethods	1	4	7	25
MLLE	**0.9521**	**0.9852**	**0.9748**	**0.9010**
LLE	0.9529	0.9558	0.9524	0.7978
MSE	0.9310	0.7977	0.7171	0.5584
LE	0.9229	0.9081	0.8809	0.5034
PCA	0.9352	0.9549	0.9416	0.7283

### Discussion

There are two reasons that make MLLE more effective to learn a low-dimensional embedding from multiview features, compared with existing dimensionality reduction methods. *The first* is that MLLE can simultaneously learn a low-dimensional embedding on multiview features. Different from other methods, MLLE uses LLE to obtain optimal low-dimensional subspace on each view and global coordinate alignment technique to unify all learned subspaces into a global one. *The second* is that MLLE can explore complementary properties among different features. Different from traditional dimensionality reduction methods that treat each feature equally, MLLE assigns different weight to each feature and utilizes alternating optimization technique to obtain these weights. Experimental results demonstrate the effectiveness of MLLE, in the context of CBMIR, compared with existing methods.

### Conclusion

With the rapid proliferation of radiological images in the medical domain, retrieving medical images from large archives to aid radiological image interpretation is becoming one of the most active research fields. CBMIR utilizes multiple visual features to represent images, which brings the problem of the “curse of dimensionality”. Though conventional dimensional reduction methods can be employed to tackle this problem, these solutions ignore the fact that different visual features have a range of physical meanings. There is therefore a challenge to discover the complementary properties of multiple visual features to represent medical images. In this paper, we propose a new multiview learning method called MLLE to address the problem. Experimental evaluations on a subset of the IRMA medical image dataset have demonstrated that the new method effectively represents medical images in a low-dimensional subspace, and thus improves the performance of CBMIR significantly.

In the proposed method, it is found that local patch size 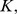 subspace dimension 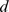 and scaling factor 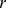 affect the effectiveness of MLLE. From **Figure 10**, **Table 9** and **Figure 11** we can see that optimal parameter values for MLLE exist on the IRMA medical image dataset. In the future, we will evaluate the performance of MLLE on other medical image test bed to further explore effects of parameters on MLLE.

## Supporting Information

Appendix S1 **Detailed Derivation of** **Equation (9** **).**
(DOC)

Appendix S2 **Proof of** ***L^v^*** **is Symmetric and Positive Semidefinite.**
(DOC)
