# Supplementary material for: Multiview Locally Linear Embedding for Effective Medical Image Retrieval
Source: PLoS One. 2013 Dec 13;8(12):e82409. doi: 10.1371/journal.pone.0082409 (PMC3862625; doi:10.1371/journal.pone.0082409)
Supplement: Appendix S1 — Detailed Derivation of Equation (9 ). (DOC) [file pone.0082409.s001.doc]

**Appendix S1**: Detailed Derivation of Equation

$$\begin{matrix} \mathop{\mathrm{argmin}}\limits_{Y,\vec{c}} \sum_{i=1}^{N} \sum_{v=1}^{V} c_{v}tr(YS_{i}^{v}L_{i}^{v}(YS_{i}^{v})^{T}) \\ =\mathop{\mathrm{argmin}}\limits_{Y,\vec{c}} \sum_{i=1}^{N} \sum_{v=1}^{V} c_{v}tr(YS_{i}^{v}L_{i}^{v}(S_{i}^{v})^{T}Y^{T}) \\ =\mathop{\mathrm{argmin}}\limits_{Y,\vec{c}} \sum_{v=1}^{V} c_{v}\sum_{i=1}^{N} tr(YS_{i}^{v}L_{i}^{v}(S_{i}^{v})^{T}Y^{T}) \\ =\mathop{\mathrm{argmin}}\limits_{Y,\vec{c}} \sum_{v=1}^{V} c_{v}tr(\sum_{i=1}^{N} YS_{i}^{v}L_{i}^{v}(S_{i}^{v})^{T}Y^{T}) \\ =\mathop{\mathrm{argmin}}\limits_{Y,\vec{c}} \sum_{v=1}^{V} c_{v}tr(Y(\sum_{i=1}^{N} S_{i}^{v}L_{i}^{v}(S_{i}^{v})^{T})Y^{T}) \\ =\mathop{\mathrm{argmin}}\limits_{Y,\vec{c}} \sum_{v=1}^{V} c_{v}tr(YL^{v}Y^{T}) \end{matrix}$$
