# Supplementary material for: Multiview Locally Linear Embedding for Effective Medical Image Retrieval
Source: PLoS One. 2013 Dec 13;8(12):e82409. doi: 10.1371/journal.pone.0082409 (PMC3862625; doi:10.1371/journal.pone.0082409)
Supplement: Appendix S2 — Proof of Lv is Symmetric and Positive Semidefinite. (DOC) [file pone.0082409.s002.doc]

**Appendix S2:** Proof of $L^{v}$is Symmetric and Positive Semidefinite

$$\begin{matrix} (L^{v})^{T}=(\sum_{i=1}^{N} S_{i}^{v}L_{i}^{v}(S_{i}^{v})^{T})^{T} \\ =\sum_{i=1}^{N} (S_{i}^{v}L_{i}^{v}(S_{i}^{v})^{T})^{T} \\ =\sum_{i=1}^{N} S_{i}^{v}(L_{i}^{v})^{T}(S_{i}^{v})^{T} \\ =\sum_{i=1}^{N} S_{i}^{v}\left[\begin{matrix} 1 & -\vec{w}_{i}^{T} \\ -\vec{w}_{i} & \vec{w}_{i}\vec{w}_{i}^{T} \end{matrix}\right]^{T}(S_{i}^{v})^{T} \\ =\sum_{i=1}^{N} S_{i}^{v}\left[\begin{matrix} 1 & -\vec{w}_{i}^{T} \\ -\vec{w}_{i} & \vec{w}_{i}\vec{w}_{i}^{T} \end{matrix}\right](S_{i}^{v})^{T} \\ =\sum_{i=1}^{N} S_{i}^{v}L_{i}^{v}(S_{i}^{v})^{T} \\ =L^{v}, \end{matrix}$$

thus$L^{v}$is symmetric.

Given a vector

$$\begin{matrix} \vec{f}^{T}L^{v}\vec{f}=\vec{f}^{T}(\sum_{i=1}^{N} S_{i}^{v}L_{i}^{v}(S_{i}^{v})^{T})\vec{f} \\ =\sum_{i=1}^{N} \vec{f}^{T}(S_{i}^{v}L_{i}^{v}(S_{i}^{v})^{T})\vec{f} \\ =\sum_{i=1}^{N} ((S_{i}^{v})^{T}\vec{f})^{T}L_{i}^{v}((S_{i}^{v})^{T}\vec{f}), \end{matrix}$$

where

Because

$$\begin{matrix} L_{i}^{v}=\left[\begin{matrix} -1 \\ \vec{w}_{i} \end{matrix}\right]\left[\begin{matrix} -1 & \vec{w}_{i}^{T} \end{matrix}\right] \\ =\left[\begin{matrix} -1 \\ \vec{w}_{i} \end{matrix}\right]\left[\begin{matrix} -1 \\ \vec{w}_{i} \end{matrix}\right]^{T}, \end{matrix}$$

so$L_{i}^{v}$is positive semidefinite,$((S_{i}^{v})^{T}\vec{f})^{T}L_{i}^{v}((S_{i}^{v})^{T}\vec{f})\geq0.$

Then

$$\begin{matrix} \vec{f}^{T}L^{v}\vec{f}=\sum_{i=1}^{N} ((S_{i}^{v})^{T}\vec{f})^{T}L_{i}^{v}((S_{i}^{v})^{T}\vec{f}) \\ \geq0, \end{matrix}$$

thus$L^{v}$is positive semidefinite.
